# Gene Expression Profiles are Altered in Human Papillomavirus-16 E6 D25E-Expressing Cell Lines

**DOI:** 10.1186/1743-422X-8-453

**Published:** 2011-09-25

**Authors:** Mi Jang, Jee Eun Rhee, Dai-Ho Jang, Sung Soon Kim

**Affiliations:** 1 Division of AIDS, Center for Immunology and Pathology, Korea National Institute of Health, Seoul, Republic of Korea

## Abstract

Previously, we have reported that the human papillomavirus (HPV) type 16 E6 D25E is the most prevalent variant in Korean women at high risk for cervical cancers. Several studies have identified an association between the increased frequency of this variant and the elevated risk of cervical intraepithelial neoplasia and invasive cervical carcinoma. To investigate whether the HPV-16 E6 D25E variant might influence cervical cancer progression, we used an oligonucleotide microarray approach to identify transcriptionally altered gene expression patterns in recombinant wild-type E6 or E6 D25E variant-expressing HPV-negative cancer cells. We found that 211 genes were significantly up- or down-regulated (at least 1.5-fold, p < 0.05). We identified 14 genes, nine down-regulated and five up-regulated upon E6 D25E expression, compared with wild-type E6 expression. These results further emphasize the unique biological activity of the HPV-16 E6 D25E variant.

## Findings

High-risk types of the human papillomavirus (HPV) are causative agents in most cases of cervical carcinoma. Much of the transforming ability of this virus can be attributed to the activities of its E6 and E7 oncoproteins [1]. The gene for E6 is one of the earliest expressed during HPV infection and plays an important role in cellular immortalization and transformation [2]. The best-known activity of E6 is its ability to accelerate the degradation of the p53 protein [3]. E6 also has multiple transformation properties such as regulating transcriptional regulators and cellular homeostasis, which are finely tuned by controlling proliferation, differentiation and apoptosis [4]. Interestingly, the E6 gene can immortalize primary human mammary epithelial cells in culture [2] and induced malignancy in a transgenic mouse model [5].

HPV-16 is the most frequent type found in invasive cervical carcinoma (ICC) as well as in cervical intraepithelial neoplasia (CIN) in Asian women. Accumulated epidemiological studies suggest that certain HPV-16 E6 variants could influence the persistence of the infection [6]. Furthermore, one HPV-16 E6 variant harboring an amino acid change at position 83 by substituting leucine for valine (L83V) was reported in Swedish and Italian populations [7,8], and another change at position 25 by substituting aspartic acid for glutamic acid (D25E) was reported in Japanese [9] and Chinese [10] populations: these were associated with the progression of cervical carcinoma. Our previous study showed that HPV-16 E6 D25E is the most prevalent variant in Korean women at high risk for developing cervical cancers [11]. However, no studies have addressed the molecular relationship between the E6 D25E variant and cervical carcinogenesis. To identify alterations in the gene regulatory network that might be associated with the presence of the HPV-16 E6 D25E compared with wild-type E6 in cervical carcinogenesis, we performed microarray analyses in HPV-negative cancer cell lines expressing recombinant wild-type E6 or E6 D25E variant proteins.

The HPV-negative cervical cancer cell line C33A was purchased from the American Type Culture Collection (Rockville, MD, USA) and maintained in modified Eagle's medium supplemented with 10% fetal bovine serum (Gibco BRL, Rockville, MD, USA), penicillin (100 U/ml) and streptomycin (100 μg/ml) at 37 °C in a humidified incubator with 5% CO_2_.

The wild-type E6 and D25E variant genes were amplified from HPV-16-infected cervical swabs [10] and inserted into a pLenti6.3/V5-TOPO lentiviral vector (Invitrogen, Carlsbad, CA, USA) followed by sequence analysis. C33A cells were infected with the recombinant lentivirus and the cells with stably maintained E6 genes were screened by blasticidin selection (10 μg/μl; Invitrogen).

To confirm the expression of the E6 genes, we performed reverse transcription polymerase chain reaction (RT-PCR) and immunofluorescence. Total cellular RNAs were isolated using RNeasy kits (QIAGEN, Hilden, Germany) and RT-PCR reactions were performed with OneStep RT-PCR kits (QIAGEN) according to the manufacturer's instructions. Primers used for amplifying the gene sequence for E6 were: forward 5"-GCAATGTTTCAGGACCCACA-3" and reverse 5"-ACAGCATATGGATTCCCATCTC-3". Primers for the GAPDH gene (as internal control) were: forward 5"-CCACAGTCCATGCCATC-3" and reverse 5"-TCCACCACCCTGTTGCTGT-3". The temperature profile comprising the RT round was 50 °C for 30 min. The PCR rounds involved denaturing at 95 °C for 15 min followed by 30 cycles of 94 °C for 30 s, 55 °C for 30 s, 72 °C for 30 s and one cycle at 72 °C for 10 min. For the immunofluorescence, cells were seeded in six-well plates, fixed at room temperature for 15 min with BD Cytofix/Cytoperm (BD Biosciences, Franklin Lakes, NJ, USA) after twenty-four hours and treated for 30 min with Image-iT FX signal enhancer (Invitrogen). Following blocking with 3% bovine serum albumin in phosphate-buffered saline for 10 min, cells were incubated with the primary antibody overnight at 4°C and with the secondary antibody for 1 h at room temperature. The primary antibody was anti-E6 (sc-460, Santa Cruz Biotechnology, Santa Cruz, CA, USA) and the secondary antibody was Alexa Fluor 546-labeled mouse immunoglobulin G (A11003, Molecular Probes, Eugene, OR, USA) (both at 1:100 dilution). Confocal imaging was performed using an LSM 510 META microscope (Carl Zeiss Microimaging GmbH, Jena, Germany). Messenger RNA expression levels for the survivin gene were analyzed via quantitative real time reverse transcription polymerase chain reaction (RT-PCR) using a Bio-Rad iCycler system (Bio-Rad, Hercules, CA, USA). Total RNA was reverse-transcribed into cDNA using an Omniscript RT kit (Qiagen). Primer specificity was tested by running a regular PCR for 40 cycles (95°C for 20 s and 60°C for 1 min), followed by agarose gel electrophoresis. Real time RT-PCR was performed using a SYBR supermix kit (Bio-Rad). Samples were subjected to 45 cycles of 95°C for 20 s and 60°C for 1 min. PCR efficiency was determined by running serial dilutions of template cDNA, and melting curve data were collected to assure PCR specificity. Each cDNA sample was analyzed in triplicate, and the corresponding no-RT mRNA sample was included as a negative control. A GAPDH primer was included in every plate as an internal loading control. The mRNA level of each sample for survivin gene was normalized against that of GAPDH mRNA. The relative mRNA level was determined as 2^[(Ct/Actin - Ct/gene of interest)]^. All data are presented as the mean ± standard deviation (SD) of three separate experiments. The following primers were used for the quantitative real time RT-PCR of survivin and actin genes: survivin forward primer 5"-GCAATGTTTCAGGACCCACA-3" and reverse primer 5"-AGGAACAGCCGAGATGACCT-3" and actin forward primer 5"-ATCTGGCACCACACCTTCTA-3" and reverse primer 5"-GGATAGCACAGCCTGGATAC-3". For microarray analysis, the synthesis of target cRNA probes and hybridization were performed from control and test RNAs using Agilent's Low RNA Input Linear Amplification kit (Agilent Technology, Santa Clara, CA, USA) according to the manufacturer's instructions. The arrays were hybridized at 65°C for 17 h using an Agilent hybridization oven. The hybridized microarrays were washed according to the manufacturer's washing protocol. The hybridized images were scanned using Agilent's DNA microarray scanner and quantified with feature extraction software. All data normalization and selection of changed genes were performed using GeneSpringGX 7.3 (Agilent Technology). Functional annotation of genes was performed according to the Gene Ontology™ Consortium (http://www.geneontology.org/index.shtml) using GeneSpringGX 7.3. Gene classification was based on searches in BioCarta (http://cgap.nci.nih.gov/Pathways/BioCarta_Pathways), GenMAPP (http://www.genmapp.org/), DAVID (http://david.abcc.ncifcrf.gov/) and Medline databases (http://www.ncbi.nlm.nih.gov/).

**Figure 1:**
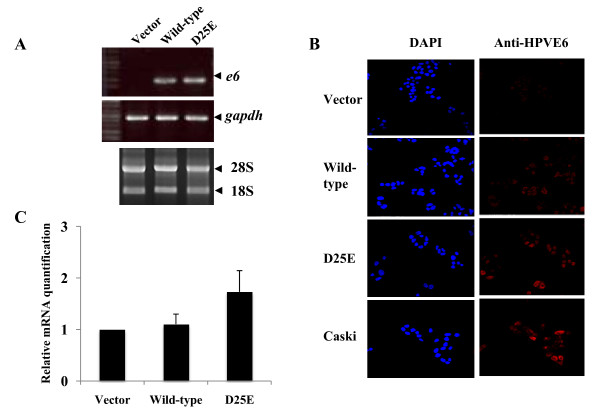
**Confirmation of HPV-16 E6 oncogenes expression and effects on the transcription of endogenous survivin gene in C33A cell lines with *e6 *oncogenes**. **A**. Quantification of mRNA of wild-type E6 and E6 D25E in C33A cells by RT-PCR performed using OneStep RT-PCR kits. GAPDH mRNA was measured as a loading control. **B**. Protein expression analysis of wild-type E6 or E6 D25E using confocal microscopy. Caski cells were used as a positive control. **C**. Quantative real-time PCR specific for survivin gene.

To investigate the role of individual viral oncoproteins of HPV-16 wild-type E6 and the D25E variant on cervical cancer progression, we stably transduced the E6 expression vector into C33A cells. Cells expressing the E6 protein were selected and maintained in medium containing blasticidin. The expression levels of E6 in C33A cells were detected by RT-PCR and immunofluorescence. As shown in Figure 1, the E6 mRNA and protein were detected in wild-type E6- or D25E variant-expressing C33A cells. In both cell lines, the expression of E6 proteins was observed at similar levels. We assessed the activation status of E6 proteins in constructed cell lines based on the mRNA level of the downstream-regulated survivin gene. HPV-16 E6 induces endogenous survivin transcription [12]. This gene is a member of the gene family involved in inhibiting apoptosis [12]. We observed that the expression of survivin was up-regulated in E6 proteins expressing C33A cells using real-time PCR (Figure 1C). This confirmed that the expressed E6 proteins were active functionally in C33A cells.

**Figure 2:**
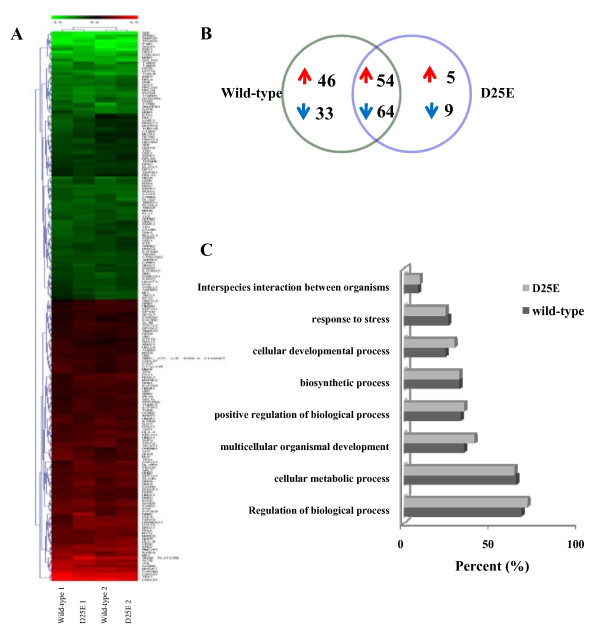
**Transcriptome analysis revealing gene expression patterns by E6 oncoproteins**. **A**. Hierarchical cluster analysis of the wild-type E6 or E6 D25E viral oncogene expression microarrays. A cluster-based representation of altered genes in E6 wild-type or E6 D25E expressed C33A with intensity provided for each cell line, normalized to the control (C33A cells not expressing E6). Genes that were up-regulated relative to control are shown in red and those that were down-regulated are green. The expression levels of these genes were altered ≥1.5-fold or ≤0.6-fold in E6-expressing cell lines compared with controls (p < 0.05). Gene symbols are shown for all in the right row. The hybridization was conducted in duplicate and denoted with 1 and 2. **B**. A venn diagram showing the numbers of genes regulated by wild-type E6 or E6 D25E. The numbers of up- or down-regulated genes that responded commonly or uniquely in response to the expression of each E6 protein are shown in red or blue arrows, respectively. The complete list of 211 genes is shown in additional file 1 and the entire microarray data set has been deposited in the Gene Ontology Consortium database (http://www.geneontology.org/index.shtml). **C**. Gene ontology-based analysis.

The HPV-16 E6 oncoprotein controls cellular gene expression in host cells during infection, and microarray technology should provide a useful experimental strategy for identifying the cellular target genes. By analyzing a genome-wide microarray, we observed significant transcriptional changes in 211 genes of the wild-type E6 or D25E protein-expressing cell lines compared with the control cell line using a filter criterion of at least 1.5-fold change with p < 0.05. There were 118 common transcripts revealed in both cell lines, 79 transcripts that were regulated by wild-type E6 and 14 transcripts that were unique in the E6 D25E-expressing cells. These results indicate that E6 might have different biological activities between variants. A hierarchical clustering heat map (Figure 2A) and stylized Venn diagram (Figure 2B) depict the patterns of changes in gene expression levels in each E6-expressing cell line. A detailed list of the gene signatures is presented in the supplemental data (Additional file 1). Functional annotation of the genes was assessed using gene ontology-based biological property analysis (DAVID; http://david.abcc.ncifcrf.gov/). The known annotations for 211 genes were submitted for level 2 biological process analyses (Figure 2C). The major gene population (>60%) was categorized as being involved in the regulation of biological and cellular metabolic processes. In addition, a minor gene population (<10%) was associated with interspecies interactions between organisms.

We have reported that E6 D25E is the most prevalent variant (68%) in Korean women at high risk for cervical cancers [11]. Epidemiological studies have shown an association of variation in the E6 genomic sequence with the progression of high-grade precursor lesions to invasive tumors [9,10,13]. The distribution of HPV 16 sequence variations in ICC was found to vary geographically [14,15]. The incidences of the variants in ICC from Japanese women were as follows: D25E in 44% of women, L83V in 28%, and other variants in 16% [9]. In China, HPV 16 D25E variant was detected in 62% of the patients with cervical cancer [10]. Additionally, HPV-16 E6 L83V is associated with an increased risk of persistent infection and cytological progression to CIN types 2 and 3 and to squamous cell carcinoma in European populations [6,7,15]. In agreement with epidemiological studies showing a link between the L83V variant and an increased risk of malignant disease, functional studies have demonstrated that the E6 L83V variant has biological advantages over E6 wild-type. One model of cervical tumor progression induced by the E6 oncogene is activation of mitogen-activated protein kinase (MAPK) signaling via the oncogenic protein Ras [16]. This effect is enhanced by the E6 L83V variant, which activates the MAPK signaling through Rap1, independent of Ras [16]. Zehbe et al. [17] evaluated functions relevant to carcinogenesis for the E6 variants L83V, R10/L83V and Q14H/H78Y/L83V as well as for the prototype. K5 and K10, markers for cell differentiation, were detected among the E6 prototype and raft cultures of variants. The E6 variants but not the prototype expressed K5 and K10 within the suprabasal compartment in all keratinocytes at the same time. Replication of suprabasal keratinocytes preserves coexpression of K5 and K10 because the premature migration of proliferating keratinocytes into the suprabasal compartment is a hallmark of early squamous cell carcinogenesis [18]. This phenotype indicates that E6 variants might be associated with promoting carcinogenesis.

Until now, there have been no experimental data on the impact of this single amino acid 25 change in prototype E6, the dominant variant in Asia region, on progression to invasive carcinomas. This study aimed at identifying genes affected by the HPV-16 E6 D25E variant in comparison with the wild-type E6 in the C33A HPV-negative cervical cancer cell line using microarrays. A genomic approach using oligonucleotide microarrays showed that 14 genes involved in interspecies interactions between organisms (including the immune system) and stress response were modulated by the E6 D25E oncogene at an mRNA level. Nine genes (*ZMZ1, RPL23, MAPK4, RPL31, RARS, LAMB3, HSPA14, AIFM2, IFRD1*) were down-regulated and five genes (*UBC, RPS9, HLA-A, -B, ROCK2*) were up-regulated in E6 D25E expressing cells. The specific functions of these genes in cervical oncogenesis remain to be elucidated. However, it is conceivable that genes such as *AIFM2, RPL23*, and *ROCK2 *could influence disease progression and promote potential carcinogenicity. In the present study, AIFM2 was reduced in E6 D25E oncoprotein expressing cell lines. It has been reported that AIFM2 has a pro-apoptotic function and that its expression is down-regulated in cancer cell lines by comparison with normal cell lines [19]. Inhibition of apoptosis is a mechanism of survival for virally or chemically transformed malignant cells. It means that E6 D25E might be involved to promote the survival of HPV-infected cells in a manner that facilitates tumor development.

We have also observed down-regulation of the *RPL23 *gene associated with cell cycle and apoptosis. RPL23 could stabilize p53, inhibiting its degradation and thus resulting in substantial cell cycle arrest at the G1-S checkpoint and/or apoptotic cell death in the cultured gastric cancer cell lines MKN45 and AGS as evidence for the antitumor activity [20]. From our data, reduced RPL23 expression might affect DNA synthesis and apoptosis inhibition through the p53 pathway. This result indicates that *RPL23 *might be directly or indirectly related to oncogenesis by E6 D25E.

In this study, based on microarray analysis, we showed that *ROCK2 *is up-regulated in E6 D25E expressing cell lines. ROCK2 kinase plays a critical role in promoting centrosome duplication and amplification [21]. In general, centrosome amplification frequently occurs in various cancers, and is considered a major cause of chromosomal instability. Overexpression of ROCK2 has also been linked frequently to enhanced metastatic potential (increase in migration and invasion of tumor cells) in various cancer types [22,23]. This increased expression of ROCK2 is indicative of a possible involvement of E6 D25E protein in the malignant transformation of cervical cells.

Our data showed that the MAPK pathway-associated genes MAPK-2, -3 and -4 were down-regulated in contrast to L83V and the expression levels of immune response-related human histocompatibility leukocyte antigen (HLA) genes HLA-A and HLA-B were elevated in E6 D25E-expressing cells. Down-regulation of HLA Class I genes (HLA-A, -B and -C) is associated with cervical neoplastic lesions and HLA polymorphisms might influence oncogenicity by HPV infection, most likely through an immunological mechanism [24]. These findings are in the conflict with the epidemical data [6,10] for oncogenic role of variant but it is still unclear whether HPV variant D25E have distinct oncogenical properties via HLA or MAPK regulation.

This is the first study to identify functional differences in the naturally occurring HPV-16 E6 D25E variant in C33A cell lines and provides a basis for future mechanistic studies. We observed that the HPV-16 E6 D25E-expressing C33A cells modulated several functional genes compared with wild-type E6-expressing cells. This result contributes new and useful information to support the epidemiological data showing that there is more frequent detection of the D25E variant in patients with cervical cancer, and that several genes such as *AIFM2*, *RPL23*, and *ROCK2 *might be involved in cervical tumorigenicity via E6 D25E. Our findings suggest that E6 D25E might have a unique oncogenic role in cancerous transformation but further studies are needed to define the molecular mechanisms and carcinogenic potential of the HPV-16 D25E variant.

## List of abbreviations

HPV: human papillomavirus; CIN: cervical intraepithelial neoplasia; RT-PCR: reverse transcription polymerase chain reaction

## Competing interests

The authors have no competing interests to declare.

## Authors' contributions

DH supported performing experiments. M and JE performed the experiments and wrote and edited the manuscript. SS designed the research and edited the manuscript. All authors read and approved the final manuscript.

## Supplementary Material

Additional file 1 **The list of genes differentially changed in C33A cells expressing E6 proteins**. The list shows genes that were significantly altered (≥1.5-fold or ≤0.6-fold, p < 0.05) in E6-expressing cell lines compared with controls. The fold change of these genes is mean of 2 independent experiments. Click here for file
