# Supplementary material for: Gene Expression Profiles are Altered in Human Papillomavirus-16 E6 D25E-Expressing Cell Lines
Source: Virol J. 2011 Sep 25;8:453. doi: 10.1186/1743-422X-8-453 (PMC3203858; doi:10.1186/1743-422X-8-453)
Supplement: Additional file 1 — The list of genes differentially changed in C33A cells expressing E6 proteins. The list shows genes that were significantly altered (≥1.5-fold or ≤0.6-fold, p < 0.05) in E6-expressing cell lines compared with controls. The fold change of these genes is mean of 2 independent experiments. [file 1743-422X-8-453-S1.DOC]

| **The list of genes differentially changed in C33A cells expressing E6 proteins** | | | | | | | | | | | |
| --- | --- | --- | --- | --- | --- | --- | --- | --- | --- | --- | --- |
|  |  |  |  |  |  | |  |  | | |  |
| **No.** | **GeneSymbol** | **Description** | **Genbank** | **UniGene** | **E6 Wild-type** | | | | **E6 D25E** | | |
|  |  |  |  |  | **Fold change** | **p-value** | | | **Fold change** | **p-value** | |
| 1 | PERP | PERP, TP53 apoptosis effector | NM_022121 | Hs.520421 | 2.08 | 0.00000057 | | | 1.39 | 0.00000037 | |
| 2 | MVP | major vault protein | NM_017458 | Hs.632177 | 2.20 | 0.01969567 | | | 1.40 | 0.00003705 | |
| 3 | HMOX1 | heme oxygenase (decycling) 1 | NM_002133 | Hs.517581 | 1.54 | 0.00003442 | | | 1.39 | 0.00000227 | |
| 4 | GJA1 | gap junction protein, alpha 1, 43kDa | NM_000165 | Hs.74471 | 1.82 | 0.00052683 | | | 1.40 | 0.00000978 | |
| 5 | YES1 | v-yes-1 Yamaguchi sarcoma viral oncogene homolog 1 | NM_005433 | Hs.194148 | 1.82 | 0.00132926 | | | 1.41 | 0.00001350 | |
| 6 | NPM1 | nucleophosmin (nucleolar phosphoprotein B23, numatrin) | NM_001037738 | Hs.557550 | 1.53 | 0.00131490 | | | 1.31 | 0.00001633 | |
| 7 | CCNG2 | cyclin G2 | NM_004354 | Hs.13291 | 1.58 | 0.00205158 | | | 1.34 | 0.00001697 | |
| 8 | NPM1 | nucleophosmin (nucleolar phosphoprotein B23, numatrin) | NM_002520 | Hs.557550 | 1.57 | 0.00019843 | | | 1.27 | 0.00002094 | |
| 9 | PPP2R5C | protein phosphatase 2, regulatory subunit B', gamma isoform | NM_178588 | Hs.368264 | 1.78 | 0.03048045 | | | 1.25 | 0.00002326 | |
| 10 | PPIG | peptidylprolyl isomerase G (cyclophilin G) | NM_004792 | Hs.470544 | 1.86 | 0.00988731 | | | 1.37 | 0.00002410 | |
| 11 | RAD21 | RAD21 homolog (S. pombe) | AW858928 | Hs.81848 | 1.66 | 0.00010157 | | | 1.29 | 0.00002650 | |
| 12 | NPM1 | nucleophosmin (nucleolar phosphoprotein B23, numatrin) | NM_001037738 | Hs.557550 | 1.50 | 0.03182312 | | | 1.38 | 0.00002698 | |
| 13 | EIF2S3 | eukaryotic translation initiation factor 2, subunit 3 gamma, 52kDa | NM_001415 | Hs.539684 | 1.65 | 0.00029272 | | | 1.30 | 0.00002921 | |
| 14 | EEF1A1 | eukaryotic translation elongation factor 1 alpha 1 | NM_001402 | Hs.586423 | 1.85 | 0.00000058 | | | 1.46 | 0.00003421 | |
| 15 | EEF1A1 | eukaryotic translation elongation factor 1 alpha 1 | NM_001402 | Hs.586423 | 1.52 | 0.01135361 | | | 1.25 | 0.00006562 | |
| 16 | SEL1L | CDNA FLJ11953 fis, clone HEMBB1000883 | AK022015 | Hs.677083 | 1.62 | 0.01439242 | | | 1.44 | 0.00006941 | |
| 17 | SP100 | SP100 nuclear antigen | NM_003113 | Hs.369056 | 1.85 | 0.00016013 | | | 1.27 | 0.00008317 | |
| 18 | EEF1A1 | Eukaryotic translation elongation factor 1 alpha 1 | XR_018189 | Hs.586423 | 1.86 | 0.00015333 | | | 1.43 | 0.00008979 | |
| 19 | MMAB | methylmalonic aciduria (cobalamin deficiency) cblB type | NM_052845 | Hs.12106 | 1.72 | 0.01703869 | | | 1.45 | 0.00009158 | |
| 20 | DPYSL2 | dihydropyrimidinase-like 2 | NM_001386 | Hs.173381 | 1.51 | 0.00677636 | | | 1.23 | 0.00013521 | |
| 21 | MAP3K2 | mitogen-activated protein kinase kinase kinase 2 | NM_006609 | Hs.145605 | 1.60 | 0.04218896 | | | 1.32 | 0.00014106 | |
| 22 | HMGB1 | High-mobility group box 1 | AF283771 | Hs.434102 | 1.97 | 0.00092819 | | | 1.25 | 0.00014360 | |
| 23 | MORC3 | MORC family CW-type zinc finger 3 | NM_015358 | Hs.421150 | 1.75 | 0.02052101 | | | 1.42 | 0.00017887 | |
| 24 | CUL5 | cullin 5 | NM_003478 | Hs.440320 | 1.97 | 0.04082028 | | | 1.49 | 0.00022768 | |
| 25 | TMED10 | transmembrane emp24-like trafficking protein 10 (yeast) | NM_006827 | Hs.74137 | 1.51 | 0.00009607 | | | 1.30 | 0.00023486 | |
| 26 | HELLS | CDNA FLJ34225 fis, clone FCBBF3023372 | BU686376 | Hs.658935 | 1.57 | 0.00061575 | | | 1.33 | 0.00034602 | |
| 27 | CYCS | cytochrome c, somatic | NM_018947 | Hs.437060 | 1.73 | 0.00003215 | | | 1.42 | 0.00041788 | |
| 28 | TCF7L2 | transcription factor 7-like 2 (T-cell specific, HMG-box) | NM_030756 | Hs.593995 | 1.51 | 0.02333122 | | | 1.44 | 0.00044084 | |
| 29 | FANCD2 | Fanconi anemia, complementation group D2 | NM_001018115 | Hs.208388 | 1.68 | 0.01815886 | | | 1.48 | 0.00047852 | |
| 30 | UBE2L3 | ubiquitin-conjugating enzyme E2L 3 | NM_003347 | Hs.108104 | 1.53 | 0.04721335 | | | 1.38 | 0.00055604 | |
| 31 | PIK3R2 | Phosphoinositide-3-kinase, regulatory subunit 2 (p85 beta) | AA042935 | Hs.371344 | 1.53 | 0.01858663 | | | 1.42 | 0.00057598 | |
| 32 | YES1 | CDNA FLJ14122 fis, clone MAMMA1002033 | AK024184 | Hs.658859 | 1.77 | 0.03689762 | | | 1.20 | 0.00081750 | |
| 33 | BIRC4 | baculoviral IAP repeat-containing 4 | NM_001167 | Hs.356076 | 1.99 | 0.00210963 | | | 1.43 | 0.00102952 | |
| 34 | POU2F1 | POU domain, class 2, transcription factor 1 | NM_002697 | Hs.493649 | 1.82 | 0.00461622 | | | 1.35 | 0.00159574 | |
| 35 | GLIPR1 | GLI pathogenesis-related 1 (glioma) | NM_006851 | Hs.205558 | 1.88 | 0.02583779 | | | 1.15 | 0.00331775 | |
| 36 | LGALS7 | lectin, galactoside-binding, soluble, 7 (galectin 7) | NM_001042507 | Hs.597642 | 1.63 | 0.00000815 | | | 1.37 | 0.00373748 | |
| 37 | SLTM | SAFB-like, transcription modulator | NM_024755 | Hs.512932 | 1.65 | 0.03953261 | | | 1.44 | 0.00437664 | |
| 38 | AFP | alpha-fetoprotein | NM_001134 | Hs.518808 | 1.75 | 0.00358323 | | | 1.40 | 0.00607674 | |
| 39 | DHX9 | DEAH (Asp-Glu-Ala-His) box polypeptide 9 | NM_001357 | Hs.191518 | 1.85 | 0.01297371 | | | 1.32 | 0.00740829 | |
| 40 | C17orf45 | chromosome 17 open reading frame 45 | NM_152350 | Hs.368934 | 1.65 | 0.00001640 | | | 1.34 | 0.00973501 | |
| 41 | CDKN2B | cyclin-dependent kinase inhibitor 2B (p15, inhibits CDK4) | NM_078487 | Hs.72901 | 1.54 | 0.00973743 | | | 0.93 | 0.01013475 | |
| 42 | STAT3 | signal transducer and activator of transcription 3 (acute-phase response factor) | NM_213662 | Hs.463059 | 1.54 | 0.00617036 | | | 1.33 | 0.01026375 | |
| 43 | SFN | stratifin | NM_006142 | Hs.523718 | 1.56 | 0.00235079 | | | 1.33 | 0.01786620 | |
| 44 | SMG1 | PI-3-kinase-related kinase SMG-1 | NM_015092 | Hs.460179 | 1.60 | 0.00022172 | | | 1.45 | 0.02266874 | |
| 45 | TWIST1 | twist homolog 1 (acrocephalosyndactyly 3; Saethre-Chotzen syndrome) (Drosophila) | NM_000474 | Hs.66744 | 1.57 | 0.00000174 | | | 1.25 | 0.03571215 | |
| 46 | HMGB1 | high-mobility group box 1 | NM_002128 | Hs.434102 | 1.91 | 0.00680191 | | | 1.20 | 0.04320334 | |
| 47 | HLA-B | Homo sapiens major histocompatibility complex, class I, B (HLA-B), mRNA [NM_005514] | NM_005514 | Hs.77961 | 1.30 | 0.00000130 | | | 1.66 | 0.00000116 | |
| 48 | UBC | Homo sapiens ubiquitin C (UBC), mRNA [NM_021009] | NM_021009 | Hs.520348 | 1.43 | 0.00017655 | | | 1.57 | 0.00013231 | |
| 49 | HLA-A | Homo sapiens, clone IMAGE:5575764, mRNA. [BC035647] | BC035647 | Hs.656882 | 1.48 | 0.00001171 | | | 1.96 | 0.00030923 | |
| 50 | ERCC1 | Homo sapiens excision repair cross-complementing rodent repair deficiency, complementation group 1 (includes overlapping antisense sequence) (ERCC1), transcript variant 2, mRNA [NM_001983] | NM_001983 | Hs.435981 | 1.42 | 0.02935504 | | | 1.53 | 0.00319050 | |
| 51 | RPS9 | Homo sapiens ribosomal protein S9 (RPS9), mRNA [NM_001013] | NM_001013 | Hs.546288 | 1.20 | 0.00382277 | | | 1.72 | 0.01000619 | |
| 52 | LGALS1 | Lectin, galactoside-binding, soluble, 1 (galectin 1) | AA627222 | Hs.445351 | 12.61 | 0.04665803 | | | 3.95 | 0.00000001 | |
| 53 | LGALS1 | lectin, galactoside-binding, soluble, 1 (galectin 1) | NM_002305 | Hs.445351 | 6.54 | 0.00593358 | | | 3.46 | 0.00000001 | |
| 54 | VHL | von Hippel-Lindau tumor suppressor | AF088066 | Hs.517792 | 4.53 | 0.00000006 | | | 3.25 | 0.00000005 | |
| 55 | CASP8 | caspase 8, apoptosis-related cysteine peptidase | NM_033355 | Hs.655983 | 3.81 | 0.04301833 | | | 2.85 | 0.00000018 | |
| 56 | PMAIP1 | phorbol-12-myristate-13-acetate-induced protein 1 | NM_021127 | Hs.96 | 2.30 | 0.00350707 | | | 1.85 | 0.00000034 | |
| 57 | CYP2B6 | cytochrome P450, family 2, subfamily B, polypeptide 6 | NM_000767 | Hs.1360 | 5.15 | 0.00000003 | | | 2.93 | 0.00000046 | |
| 58 | CDH15 | cadherin 15, M-cadherin (myotubule) | NM_004933 | Hs.148090 | 2.17 | 0.01157054 | | | 2.10 | 0.00000046 | |
| 59 | ENO2 | enolase 2 (gamma, neuronal) | NM_001975 | Hs.511915 | 2.74 | 0.00245774 | | | 2.39 | 0.00000077 | |
| 60 | HSP90AA1 | heat shock protein 90kDa alpha (cytosolic), class A member 1 | NM_001040141 | Hs.525600 | 2.19 | 0.02894250 | | | 1.85 | 0.00000088 | |
| 61 | MYT1 | myelin transcription factor 1 | NM_004535 | Hs.279562 | 2.01 | 0.00002007 | | | 1.82 | 0.00000213 | |
| 62 | HIF1A | Hypoxia-inducible factor 1, alpha subunit (basic helix-loop-helix transcription factor) | BG108194 | Hs.654600 | 2.38 | 0.02345428 | | | 1.94 | 0.00000272 | |
| 63 | ARL2 | ADP-ribosylation factor-like 2 | NM_001667 | Hs.502836 | 2.00 | 0.00212225 | | | 1.67 | 0.00000305 | |
| 64 | ACTB | actin, beta | NM_001101 | Hs.520640 | 5.06 | 0.00000007 | | | 4.55 | 0.00000367 | |
| 65 | PIK3CD | phosphoinositide-3-kinase, catalytic, delta polypeptide | NM_005026 | Hs.518451 | 2.38 | 0.00000502 | | | 1.90 | 0.00000469 | |
| 66 | RARA | retinoic acid receptor, alpha | NM_001024809 | Hs.654583 | 1.96 | 0.00000246 | | | 1.73 | 0.00000514 | |
| 67 | FANCC | Fanconi anemia, complementation group C | BC034271 | Hs.672362 | 2.57 | 0.00000073 | | | 1.96 | 0.00000595 | |
| 68 | HMGA1 | high mobility group AT-hook 1 | NM_145904 | Hs.518805 | 1.72 | 0.00000971 | | | 1.92 | 0.00000902 | |
| 69 | BRCA1 | breast cancer 1, early onset | NM_007295 | Hs.194143 | 2.54 | 0.01510021 | | | 1.88 | 0.00000926 | |
| 70 | TUBB | tubulin, beta | NM_178014 | Hs.636480 | 1.70 | 0.01033348 | | | 2.18 | 0.00001302 | |
| 71 | CAV1 | caveolin 1, caveolae protein, 22kDa | NM_001753 | Hs.74034 | 1.51 | 0.00776766 | | | 1.58 | 0.00002285 | |
| 72 | UBC | ubiquitin C | NM_021009 | Hs.520348 | 1.60 | 0.00020160 | | | 1.76 | 0.00003227 | |
| 73 | DAND5 | DAN domain family, member 5 | NM_152654 | Hs.331981 | 2.25 | 0.00000075 | | | 2.08 | 0.00003426 | |
| 74 | TFPI | Tissue factor pathway inhibitor (lipoprotein-associated coagulation inhibitor) | BE672039 | Hs.516578 | 6.19 | 0.00961239 | | | 2.29 | 0.00003749 | |
| 75 | FBXO5 | F-box protein 5 | NM_012177 | Hs.520506 | 1.95 | 0.01547506 | | | 1.65 | 0.00003939 | |
| 76 | HMGN2 | high-mobility group nucleosomal binding domain 2 | NM_005517 | Hs.181163 | 1.74 | 0.00578196 | | | 1.50 | 0.00004420 | |
| 77 | ROCK2 | Rho-associated, coiled-coil containing protein kinase 2 | NM_004850 | Hs.591600 | 1.55 | 0.04050397 | | | 1.71 | 0.00004681 | |
| 78 | RND2 | Rho family GTPase 2 | NM_005440 | Hs.603111 | 2.27 | 0.00018270 | | | 2.03 | 0.00004723 | |
| 79 | PXN | paxillin | BC052611 | Hs.661210 | 2.25 | 0.00000099 | | | 1.67 | 0.00005167 | |
| 80 | POLK | polymerase (DNA directed) kappa | NM_016218 | Hs.135756 | 2.03 | 0.00017054 | | | 1.88 | 0.00006992 | |
| 81 | TUBB2A | tubulin, beta 2A | NM_001069 | Hs.654543 | 1.59 | 0.02248840 | | | 1.89 | 0.00007010 | |
| 82 | TUBB3 | tubulin, beta 3 | NM_006086 | Hs.511743 | 1.77 | 0.00005237 | | | 1.87 | 0.00009994 | |
| 83 | DRAM | damage-regulated autophagy modulator | NM_018370 | Hs.525634 | 2.79 | 0.01964949 | | | 2.01 | 0.00010152 | |
| 84 | BPNT1 | 3'(2'), 5'-bisphosphate nucleotidase 1 | NM_006085 | Hs.406134 | 1.50 | 0.01757998 | | | 1.56 | 0.00011676 | |
| 85 | MAP4 | microtubule-associated protein 4 | NM_002375 | Hs.517949 | 1.74 | 0.00047471 | | | 1.51 | 0.00025196 | |
| 86 | BIRC6 | baculoviral IAP repeat-containing 6 (apollon) | NM_016252 | Hs.150107 | 1.57 | 0.03191659 | | | 1.54 | 0.00032293 | |
| 87 | CASP6 | caspase 6, apoptosis-related cysteine peptidase | NM_001226 | Hs.654616 | 2.22 | 0.00000133 | | | 1.58 | 0.00043271 | |
| 88 | MET | met proto-oncogene (hepatocyte growth factor receptor) | NM_000245 | Hs.132966 | 2.44 | 0.00234231 | | | 1.58 | 0.00043396 | |
| 89 | GNAS | GNAS complex locus | NM_001077489 | Hs.125898 | 2.44 | 0.03220152 | | | 1.65 | 0.00054893 | |
| 90 | NGFR | nerve growth factor receptor (TNFR superfamily, member 16) | NM_002507 | Hs.415768 | 2.25 | 0.00000540 | | | 2.14 | 0.00071126 | |
| 91 | UBC | ubiquitin C | NM_021009 | Hs.520348 | 1.63 | 0.00090617 | | | 1.81 | 0.00087319 | |
| 92 | RRM2 | ribonucleotide reductase M2 polypeptide | NM_001034 | Hs.226390 | 1.76 | 0.00185358 | | | 1.77 | 0.00122096 | |
| 93 | EZH2 | CDNA FLJ13754 fis, clone PLACE3000362 | AK023816 | Hs.659984 | 2.12 | 0.00098235 | | | 1.71 | 0.00135956 | |
| 94 | PLAUR | plasminogen activator, urokinase receptor | NM_001005377 | Hs.466871 | 2.28 | 0.00141296 | | | 1.58 | 0.00198574 | |
| 95 | MAP2K7 | mitogen-activated protein kinase kinase 7 | BC005365 |  | 4.33 | 0.00000037 | | | 2.21 | 0.00345877 | |
| 96 | RNASEH2A | ribonuclease H2, subunit A | NM_006397 | Hs.532851 | 1.62 | 0.00101658 | | | 1.74 | 0.00347712 | |
| 97 | EIF4A1 | eukaryotic translation initiation factor 4A, isoform 1 | NM_001416 | Hs.129673 | 1.77 | 0.00711215 | | | 1.67 | 0.00473835 | |
| 98 | SUFU | suppressor of fused homolog (Drosophila) | NM_016169 | Hs.404089 | 1.90 | 0.02099477 | | | 1.69 | 0.00869505 | |
| 99 | RPS11 | Ribosomal protein S11 | THC2586367 | Hs.433529 | 1.58 | 0.00039827 | | | 1.55 | 0.00883055 | |
| 100 | CHRNB1 | cholinergic receptor, nicotinic, beta 1 (muscle) | NM_000747 | Hs.330386 | 1.53 | 0.01840624 | | | 1.51 | 0.01179874 | |
| 101 | GNAS | GNAS complex locus | NM_001077489 | Hs.125898 | 2.39 | 0.03366389 | | | 1.66 | 0.02198075 | |
| 102 | SCYL1 | SCY1-like 1 (S. cerevisiae) | NM_001048218 | Hs.238839 | 1.56 | 0.03477664 | | | 1.53 | 0.03029371 | |
| 103 | CAPZA1 | capping protein (actin filament) muscle Z-line, alpha 1 | NM_006135 | Hs.514934 | 2.47 | 0.02725555 | | | 1.64 | 0.03682292 | |
| 104 | ANXA2 | 3' similar to contains Alu repetitive element;contains element LTR10 repetitive element ;, mRNA sequence. | THC2553569 | Hs.511605 | 1.77 | 0.00000601 | | | 1.71 | 0.03808984 | |
| 105 | RBMX | RNA binding motif protein, X-linked | NM_002139 | Hs.380118 | 2.40 | 0.04420248 | | | 1.55 | 0.04999553 | |
| 106 | EPHX1 | epoxide hydrolase 1, microsomal (xenobiotic) | NM_000120 | Hs.89649 | 0.59 | 0.00001016 | | | 0.81 | 0.00000125 | |
| 107 | CYR61 | cysteine-rich, angiogenic inducer, 61 | NM_001554 | Hs.8867 | 0.47 | 0.00000761 | | | 0.98 | 0.00000736 | |
| 108 | BCL2L1 | BCL2-like 1 | Z21831 | Hs.516966 | 0.52 | 0.00000555 | | | 0.67 | 0.00001079 | |
| 109 | PSEN2 | presenilin 2 (Alzheimer disease 4) | NM_000447 | Hs.25363 | 0.61 | 0.00001570 | | | 0.69 | 0.00001330 | |
| 110 | EIF3S9 | eukaryotic translation initiation factor 3, subunit 9 eta, 116kDa | NM_001037283 | Hs.371001 | 0.55 | 0.00010484 | | | 0.77 | 0.00002204 | |
| 111 | ATP6V0E2 | ATPase, H+ transporting V0 subunit e2 | NM_145230 | Hs.556998 | 0.57 | 0.00004489 | | | 0.78 | 0.00003312 | |
| 112 | RPS2 | ribosomal protein S2 | BC020336 | Hs.498569 | 0.63 | 0.00005289 | | | 0.85 | 0.00003426 | |
| 113 | SIRT2 | sirtuin (silent mating type information regulation 2 homolog) 2 (S. cerevisiae) | NM_012237 | Hs.466693 | 0.56 | 0.00001524 | | | 0.93 | 0.00007877 | |
| 114 | C1QBP | complement component 1, q subcomponent binding protein | NM_001212 | Hs.555866 | 0.56 | 0.04593591 | | | 0.72 | 0.00010294 | |
| 115 | ODC1 | ornithine decarboxylase 1 | NM_002539 | Hs.467701 | 0.66 | 0.00014982 | | | 0.72 | 0.00010584 | |
| 116 | SEPHS1 | selenophosphate synthetase 1 | NM_012247 | Hs.124027 | 0.66 | 0.04276975 | | | 0.75 | 0.00011908 | |
| 117 | RFT1 | RFT1 homolog (S. cerevisiae) | NM_052859 | Hs.631910 | 0.66 | 0.03103147 | | | 0.71 | 0.00012556 | |
| 118 | SRF | serum response factor (c-fos serum response element-binding transcription factor) | NM_003131 | Hs.520140 | 0.58 | 0.00001719 | | | 0.78 | 0.00014923 | |
| 119 | EIF3S12 | eukaryotic translation initiation factor 3, subunit 12 | NM_013234 | Hs.314359 | 0.61 | 0.00136093 | | | 0.70 | 0.00032043 | |
| 120 | IGFBP2 | insulin-like growth factor binding protein 2, 36kDa | NM_000597 | Hs.438102 | 0.51 | 0.00487949 | | | 0.81 | 0.00042769 | |
| 121 | DCTN2 | dynactin 2 (p50) | NM_006400 | Hs.289123 | 0.53 | 0.00439969 | | | 0.78 | 0.00057929 | |
| 122 | YKT6 | YKT6 v-SNARE homolog (S. cerevisiae) | NM_006555 | Hs.520794 | 0.66 | 0.00038258 | | | 0.83 | 0.00070860 | |
| 123 | MDM1 | Mdm4, transformed 3T3 cell double minute 1, p53 binding protein (mouse) | NM_020128 | Hs.655702 | 0.63 | 0.04521383 | | | 0.83 | 0.00076306 | |
| 124 | RPL10 | ribosomal protein L10 | NM_006013 | Hs.534404 | 0.60 | 0.04248108 | | | 0.80 | 0.00269244 | |
| 125 | FLII | flightless I homolog (Drosophila) | NM_002018 | Hs.513984 | 0.48 | 0.00001393 | | | 0.68 | 0.00310081 | |
| 126 | MCTS1 | malignant T cell amplified sequence 1 | NM_014060 | Hs.102696 | 0.59 | 0.00004718 | | | 0.70 | 0.00313593 | |
| 127 | POLR1D | polymerase (RNA) I polypeptide D, 16kDa | NM_015972 | Hs.507584 | 0.57 | 0.00134933 | | | 0.79 | 0.00563305 | |
| 128 | RPL29 | ribosomal protein L29 | NM_000992 | Hs.425125 | 0.65 | 0.00042051 | | | 0.84 | 0.00632682 | |
| 129 | CCRK | cell cycle related kinase | NM_001039803 | Hs.522274 | 0.56 | 0.03744345 | | | 0.67 | 0.00812620 | |
| 130 | EIF3S12 | eukaryotic translation initiation factor 3, subunit 12 | NM_013234 | Hs.314359 | 0.66 | 0.00006374 | | | 0.83 | 0.00837733 | |
| 131 | MLL | myeloid/lymphoid or mixed-lineage leukemia (trithorax homolog, Drosophila) | AF487905 | Hs.258855 | 0.58 | 0.02476369 | | | 0.81 | 0.00896595 | |
| 132 | ARRB2 | arrestin, beta 2 | NM_004313 | Hs.435811 | 0.62 | 0.00017257 | | | 0.83 | 0.01723727 | |
| 133 | RPL35 | ribosomal protein L35 | NM_007209 | Hs.182825 | 0.56 | 0.00014579 | | | 0.75 | 0.01874007 | |
| 134 | CHKA | choline kinase alpha | NM_001277 | Hs.569019 | 0.59 | 0.01109181 | | | 0.78 | 0.02676375 | |
| 135 | ST5 | suppression of tumorigenicity 5 | NM_005418 | Hs.117715 | 0.55 | 0.00006828 | | | 0.67 | 0.03216237 | |
| 136 | SSRP1 | structure specific recognition protein 1 | NM_003146 | Hs.523680 | 0.63 | 0.00018126 | | | 0.75 | 0.03468745 | |
| 137 | TUBA1B | Tubulin, alpha 1b | AI608782 | Hs.524390 | 0.52 | 0.03802564 | | | 0.87 | 0.03746864 | |
| 138 | CABC1 | chaperone, ABC1 activity of bc1 complex homolog (S. pombe) | NM_020247 | Hs.118241 | 0.61 | 0.04874228 | | | 1.11 | 0.04081160 | |
| 139 | ZMIZ1 | zinc finger, MIZ-type containing 1 | NM_020338 | Hs.193118 | 0.97 | 0.01247091 | | | 0.57 | 0.00003286 | |
| 140 | RPL23 | ribosomal protein L23 | NM_000978 | Hs.406300 | 0.72 | 0.00798432 | | | 0.63 | 0.00081023 | |
| 141 | MAP4K4 | mitogen-activated protein kinase kinase kinase kinase 4 | NM_145686 | Hs.431550 | 0.72 | 0.03629133 | | | 0.64 | 0.00105176 | |
| 142 | RPL31 | ribosomal protein L31 | NM_000993 | Hs.469473 | 0.78 | 0.00592411 | | | 0.66 | 0.00213263 | |
| 143 | RARS | arginyl-tRNA synthetase | NM_002887 | Hs.654907 | 0.85 | 0.01101853 | | | 0.65 | 0.00242131 | |
| 144 | LAMB3 | laminin, beta 3 | NM_001017402 | Hs.497636 | 0.86 | 0.01465433 | | | 0.62 | 0.00436670 | |
| 145 | HSPA14 | heat shock 70kDa protein 14 | NM_016299 | Hs.534169 | 0.67 | 0.00001407 | | | 0.61 | 0.00563999 | |
| 146 | IFRD1 | interferon-related developmental regulator 1 | NM_001007245 | Hs.7879 | 0.69 | 0.00135017 | | | 0.67 | 0.02038579 | |
| 147 | AIFM2 | apoptosis-inducing factor, mitochondrion-associated, 2 | NM_032797 | Hs.655377 | 1.21 | 0.00902236 | | | 0.49 | 0.02326294 | |
| 148 | KRT18 | keratin 18 | L32537 | Hs.406013 | 0.37 | 0.00000157 | | | 0.66 | 0.00000000 | |
| 149 | TFAP2C | Transcription factor AP-2 gamma (activating enhancer binding protein 2 gamma) | CB305813 | Hs.473152 | 0.19 | 0.00027196 | | | 0.22 | 0.00000000 | |
| 150 | SGK | serum/glucocorticoid regulated kinase | NM_005627 | Hs.510078 | 0.13 | 0.01564712 | | | 0.32 | 0.00000001 | |
| 151 | ENC1 | ectodermal-neural cortex (with BTB-like domain) | NM_003633 | Hs.104925 | 0.34 | 0.01732159 | | | 0.14 | 0.00000006 | |
| 152 | HES1 | hairy and enhancer of split 1, (Drosophila) | NM_005524 | Hs.250666 | 0.27 | 0.00996709 | | | 0.30 | 0.00000008 | |
| 153 | PIM1 | pim-1 oncogene | NM_002648 | Hs.81170 | 0.18 | 0.00000003 | | | 0.22 | 0.00000011 | |
| 154 | SMARCB1 | SWI/SNF related, matrix associated, actin dependent regulator of chromatin, subfamily b, member 1 | NM_003073 | Hs.534350 | 0.63 | 0.00008537 | | | 0.63 | 0.00000027 | |
| 155 | TIMP3 | TIMP metallopeptidase inhibitor 3 (Sorsby fundus dystrophy, pseudoinflammatory) | NM_000362 | Hs.644633 | 0.26 | 0.00018383 | | | 0.28 | 0.00000038 | |
| 156 | RPS2 | ribosomal protein S2 | BC020336 | Hs.498569 | 0.40 | 0.00000055 | | | 0.54 | 0.00000065 | |
| 157 | FOXO3 | Forkhead box O3 | CA420688 | Hs.220950 | 0.47 | 0.00000177 | | | 0.36 | 0.00000068 | |
| 158 | COL1A2 | collagen, type I, alpha 2 | NM_000089 | Hs.489142 | 0.44 | 0.00667412 | | | 0.26 | 0.00000103 | |
| 159 | PODXL | podocalyxin-like | NM_001018111 | Hs.16426 | 0.56 | 0.00003382 | | | 0.54 | 0.00000121 | |
| 160 | ERBB2 | v-erb-b2 erythroblastic leukemia viral oncogene homolog 2, neuro/glioblastoma derived oncogene homolog (avian) | NM_001005862 | Hs.446352 | 0.12 | 0.02746923 | | | 0.19 | 0.00000191 | |
| 161 | TRRAP | transformation/transcription domain-associated protein | NM_003496 | Hs.203952 | 0.56 | 0.00000203 | | | 0.60 | 0.00000226 | |
| 162 | RPL28 | ribosomal protein L28 | NM_000991 | Hs.652114 | 0.36 | 0.01765548 | | | 0.58 | 0.00000227 | |
| 163 | ODC1 | Ornithine decarboxylase 1 | BG462058 | Hs.467701 | 0.59 | 0.00000016 | | | 0.63 | 0.00000254 | |
| 164 | SAFB | scaffold attachment factor B | NM_002967 | Hs.23978 | 0.45 | 0.00066567 | | | 0.41 | 0.00000312 | |
| 165 | ELAVL1 | ELAV (embryonic lethal, abnormal vision, Drosophila)-like 1 (Hu antigen R) | NM_001419 | Hs.184492 | 0.66 | 0.00132723 | | | 0.61 | 0.00000432 | |
| 166 | ENO1 | Enolase 1, (alpha) | BC005884 | Hs.517145 | 0.39 | 0.01099005 | | | 0.57 | 0.00000437 | |
| 167 | IMPDH1 | IMP (inosine monophosphate) dehydrogenase 1 | NM_000883 | Hs.654401 | 0.54 | 0.00001142 | | | 0.66 | 0.00000683 | |
| 168 | TMEPAI | transmembrane, prostate androgen induced RNA | NM_020182 | Hs.517155 | 0.48 | 0.00014185 | | | 0.49 | 0.00000779 | |
| 169 | ID1 | inhibitor of DNA binding 1, dominant negative helix-loop-helix protein | NM_002165 | Hs.504609 | 0.56 | 0.00357505 | | | 0.54 | 0.00001391 | |
| 170 | HSPA8 | Heat shock 70kDa protein 8 | BU731317 | Hs.180414 | 0.64 | 0.00000130 | | | 0.57 | 0.00001573 | |
| 171 | HDAC7A | histone deacetylase 7A | NM_015401 | Hs.200063 | 0.65 | 0.00038754 | | | 0.60 | 0.00003672 | |
| 172 | PHB | prohibitin | NM_002634 | Hs.514303 | 0.39 | 0.00000021 | | | 0.59 | 0.00003909 | |
| 173 | MDM1 | Mdm4, transformed 3T3 cell double minute 1, p53 binding protein (mouse) | NM_017440 | Hs.655702 | 0.31 | 0.00001570 | | | 0.37 | 0.00004164 | |
| 174 | ECT2 | epithelial cell transforming sequence 2 oncogene | NM_018098 | Hs.518299 | 0.66 | 0.00630319 | | | 0.57 | 0.00007344 | |
| 175 | TDG | thymine-DNA glycosylase | NM_003211 | Hs.584809 | 0.65 | 0.00001646 | | | 0.57 | 0.00009189 | |
| 176 | PXN | Paxillin | CR626729 | Hs.446336 | 0.48 | 0.01364180 | | | 0.51 | 0.00010874 | |
| 177 | PRDX4 | peroxiredoxin 4 | NM_006406 | Hs.83383 | 0.56 | 0.00001272 | | | 0.64 | 0.00011694 | |
| 178 | TXNRD1 | Transcribed locus, strongly similar to XP_001060296.1 similar to ribosomal protein L18a [Rattus norvegicus] | CB114618 | Hs.567352 | 0.35 | 0.00556006 | | | 0.51 | 0.00012347 | |
| 179 | NTN1 | CDNA clone IMAGE:30530513 | BC092429 | Hs.660885 | 0.62 | 0.00102479 | | | 0.59 | 0.00014028 | |
| 180 | KRT8 | keratin 8 | NM_002273 | Hs.533782 | 0.46 | 0.00127504 | | | 0.45 | 0.00020294 | |
| 181 | GADD45G | growth arrest and DNA-damage-inducible, gamma | NM_006705 | Hs.9701 | 0.50 | 0.00000899 | | | 0.62 | 0.00029375 | |
| 182 | RPS27A | Ribosomal protein S27a | THC2587773 | Hs.311640 | 0.60 | 0.04169942 | | | 0.56 | 0.00052227 | |
| 183 | NR2C1 | nuclear receptor subfamily 2, group C, member 1 | NM_001032287 | Hs.108301 | 0.54 | 0.00052019 | | | 0.60 | 0.00054083 | |
| 184 | TAGLN | transgelin | NM_001001522 | Hs.632099 | 0.53 | 0.00578492 | | | 0.46 | 0.00078636 | |
| 185 | GAS1 | growth arrest-specific 1 | NM_002048 | Hs.65029 | 0.43 | 0.00002624 | | | 0.51 | 0.00100337 | |
| 186 | NEU3 | sialidase 3 (membrane sialidase) | NM_006656 | Hs.191074 | 0.51 | 0.00800299 | | | 0.49 | 0.00119719 | |
| 187 | PDPK1 | 3-phosphoinositide dependent protein kinase-1 | NM_002613 | Hs.459691 | 0.54 | 0.00038541 | | | 0.65 | 0.00133660 | |
| 188 | IL8 | Interleukin 8 | AJ519285 | Hs.551925 | 0.60 | 0.00612620 | | | 0.47 | 0.00158751 | |
| 189 | CSNK2A1 | casein kinase 2, alpha 1 polypeptide | NM_177559 | Hs.644056 | 0.60 | 0.00044675 | | | 0.66 | 0.00164607 | |
| 190 | HEATR1 | HEAT repeat containing 1 | NM_018072 | Hs.652333 | 0.54 | 0.00001636 | | | 0.55 | 0.00190609 | |
| 191 | BTG1 | B-cell translocation gene 1, anti-proliferative | THC2575688 | Hs.255935 | 0.44 | 0.00000635 | | | 0.62 | 0.00228556 | |
| 192 | DYNLL1 | CDNA: FLJ23130 fis, clone LNG08419 | BC053632 | Hs.660080 | 0.61 | 0.01283982 | | | 0.61 | 0.00319552 | |
| 193 | YWHAQ | Tyrosine 3-monooxygenase/tryptophan 5-monooxygenase activation protein, theta polypeptide | BG989839 | Hs.74405 | 0.59 | 0.00000300 | | | 0.59 | 0.00405592 | |
| 194 | SRRM2 | serine/arginine repetitive matrix 2 | NM_016333 | Hs.693608 | 0.60 | 0.00071445 | | | 0.61 | 0.00423654 | |
| 195 | TP53RK | TP53 regulating kinase | NM_033550 | Hs.440263 | 0.65 | 0.00091342 | | | 0.65 | 0.00582436 | |
| 196 | MSH6 | mutS homolog 6 (E. coli) | NM_000179 | Hs.445052 | 0.56 | 0.00000837 | | | 0.65 | 0.00665117 | |
| 197 | SOCS1 | suppressor of cytokine signaling 1 | NM_003745 | Hs.50640 | 0.39 | 0.00001064 | | | 0.26 | 0.00693033 | |
| 198 | CD36 | CD36 molecule (thrombospondin receptor) | NM_001001547 | Hs.120949 | 0.24 | 0.00312814 | | | 0.27 | 0.00718388 | |
| 199 | RPL4 | Ribosomal protein L4 | THC2537928 | Hs.186350 | 0.38 | 0.00000019 | | | 0.45 | 0.01034359 | |
| 200 | RPL7 | Ribosomal protein L7 | THC2575761 | Hs.421257 | 0.60 | 0.00001198 | | | 0.49 | 0.01183236 | |
| 201 | SEMA3F | sema domain, immunoglobulin domain (Ig), short basic domain, secreted, (semaphorin) 3F | NM_004186 | Hs.32981 | 0.66 | 0.00000093 | | | 0.30 | 0.01392518 | |
| 202 | SIVA1 | SIVA1, apoptosis-inducing factor | AA427821 | Hs.112058 | 0.51 | 0.00144642 | | | 0.60 | 0.01590505 | |
| 203 | PLAGL2 | Pleiomorphic adenoma gene-like 2 | AA878126 | Hs.154104 | 0.49 | 0.00000147 | | | 0.62 | 0.01664133 | |
| 204 | FGFR1 | Transcribed locus, moderately similar to XP_001085153.1 similar to 40S ribosomal protein S20 isoform 1 [Macaca mulatta] | CA427417 | Hs.675819 | 0.57 | 0.00000872 | | | 0.57 | 0.01773082 | |
| 205 | OLIG2 | oligodendrocyte lineage transcription factor 2 | NM_005806 | Hs.176977 | 0.61 | 0.00611817 | | | 0.50 | 0.02491691 | |
| 206 | TIMP3 | TIMP metallopeptidase inhibitor 3 (Sorsby fundus dystrophy, pseudoinflammatory) | X77690 | Hs.644633 | 0.38 | 0.02254410 | | | 0.36 | 0.02563773 | |
| 207 | UBE2I | Ubiquitin-conjugating enzyme E2I (UBC9 homolog, yeast) | BC064492 | Hs.302903 | 0.47 | 0.03448248 | | | 0.56 | 0.03085182 | |
| 208 | SMO | smoothened homolog (Drosophila) | NM_005631 | Hs.437846 | 0.55 | 0.00002080 | | | 0.63 | 0.03281317 | |
| 209 | RPL23A | Ribosomal protein L23a | THC2509970 | Hs.419463 | 0.40 | 0.00015668 | | | 0.40 | 0.03761329 | |
| 210 | CXADR | coxsackie virus and adenovirus receptor | NM_001338 | Hs.693697 | 0.62 | 0.02327955 | | | 0.57 | 0.04023092 | |
| 211 | CABLES1 | Cdk5 and Abl enzyme substrate 1 | NM_138375 | Hs.11108 | 0.24 | 0.00003556 | | | 0.37 | 0.04655602 | |
